# Therapeutic Implications of Immunogenic Cell Death in Human Cancer

**DOI:** 10.3389/fimmu.2013.00503

**Published:** 2014-01-06

**Authors:** Fabio Palombo, Chiara Focaccetti, Vincenzo Barnaba

**Affiliations:** ^1^Dipartimento di Medicina Interna e Specialità Mediche, Sapienza Università di Roma, Rome, Italy; ^2^Istituto Pasteur – Fondazione Cenci Bolognetti, Rome, Italy

**Keywords:** dendritic cells, T cells, chemotherapy, immunogenic cell death, tumor antigens

## Abstract

Dendritic cells (DCs) are central to the adoptive immune response, and their function is regulated by diverse signals in a context-specific manner. Different DCs have been described in physiologic conditions, inflammation, and cancer, prompting a series of questions on how adoptive immune responses, or tolerance, develop against tumors. Increasing evidence suggests that tumor treatments induce a dramatic change on tumor-infiltrating lymphocytes and, in particular, on some DC subtypes. In this review, we summarize the latest evidence on the role of DCs in cancer and preliminary evidence on chemotherapy-associated antigens identified in human cancers.

## Introduction

Cancer is characterized not only by abnormal cell growth but also by increased and diverse modality of cell death, which is sensed by innate immune cells including macrophages and dendritic cells (DCs). Dying cells can trigger either tissue homeostatic clearance by macrophages or processing by DCs, which can integrate signals from dying cells for presentation to T cells in an immunogenic or tolerogenic manner. It is well known that antigen-specific CD4^+^ and CD8^+^ T cells are detected in advanced tumor stages and that adoptive T-cell transfer can be very effective in cancer therapy (1).

Dendritic cells are essential in priming T-cell responses upon the processing and presentation of both exogenous antigens, which are preferentially presented on major histocompatibility complex (MHC) class II molecules to CD4^+^ T cells, and endogenous antigens, which are preferentially presented on MHC class I molecules to CD8^+^ T cells. The capacity of DCs to present exogenous antigens derived from other cells (usually necrotic or apoptotic cells) or soluble antigens on class I molecules is defined as cross-presentation (2, 3). Different types of DCs have been described according to different parameters, including where they are located, the type of antigen they present, and their ability to present antigens to T cells (4). From a simplified view, DCs travel in periphery tissues in search of potential antigens that are derived from pathogen-infected cells (foreign antigens); cancer cells re-expressing developmental antigens, for which the immune tolerance is low; or cancer cells expressing mutated proteins as a consequence of the oncologic process. They are attracted in inflamed tissue by metabolic products of cell death such as ATP, or they can be guided by chemokines secreted by innate immune cells such as macrophages. During the journey, they are characterized as having a high phagocytic capacity and a low antigen-presenting capacity: this status is referred to as an immature state. Different stimuli associated with bacterial or viral infections or damage signals can then activate DCs. In lymphoid tissues, DCs present antigens to B and T cells to initiate an adaptive immune response, depending on the presence of mature signals that direct adoptive responses.

Pathogen-associated molecular patterns (PAMPs) were first postulated by Janeway (5) and then identified in different species including insects. At the core of Janeway’s hypothesis was the idea that similar structures are shared by different pathogens and that immune receptors [pattern recognition receptors (PRRs)] expressed by several types of innate immune cells have evolved to recognize them. On the same line of reasoning, it became clear that immune cells can be activated by damage (danger) signals, which share the properties of being undetectable to immune sensors during physiologic processes and being detectable in cases of injury. With a few exceptions (e.g., the association of cervix tumors with the papillomavirus, or of hepatocellular carcinoma with hepatitis B or C viruses), most tumors deregulate cell life usually in the absence of a non-self signal however, they can activate immune responses through danger signals that are referred to as, in analogy with PAMPs, damage-associated molecular patterns (DAMPs).

Dendritic cells carry out several complex tasks including antigen sampling in the periphery, cell maturation in the spleen and lymph nodes, and the critical decision-making process between immunity and tolerance (lack of immune response). These tasks are executed by DCs through a remarkable plasticity and an ability to integrate signals from a variety of receptors sensing extracellular and intracellular environments. This sophisticated system likely evolved in vertebrates as a way in which to avoid autoimmune diseases mediated by adaptive immunity; however, it can limit an effective immune response against tumors, which derive from the self.

## Human and Mouse DCs in Physiology and Cancer

Human and mouse DCs are classified as classical DCs (cDCs) and plasmacytoid DCs (pDCs) (6) and present different morphologies: pDCs are round shaped, whereas cDCs have dendrites, distinct membrane markers, and different functions, and derive from different precursors within the myeloid lineage. This intricately connected system has made it difficult to distinguish DCs from other myeloid cells. To resolve this issue in mice, DC subtypes have been characterized through genetic ablation of key genes, transfer of purified cells, and functional studies. Traditionally, cDCs have been identified in mice by CD11c expression (7). However, depletion of cells expressing this marker resulted in ablation of not only cDCs but also pDCs (8). To obtain a more precise picture of DC populations, lineage-specific transcription factors have been identified [reviewed in Ref. (4)]. Two transcription factors, Flt3 and Xcr1, are associated with murine DCs, but not with macrophages, which is in line with their function during DC development. However, expression of transcription factors can be tissue specific. For instance, Zbtb46 distinguishes cDCs from other myeloid and lymphoid cells, but it is downregulated after DC stimulation; it is also found on endothelial cells, early erythroid progenitors, and monocytes stimulated with granulocyte macrophage-colony stimulating factor (GM-CSF) and interleukin 4 (IL-4). Mouse cDCs in lymphoid tissues are divided into CD8^+^ and CD4^+^ T cells and functionally classified according to antigen presentation on MHC class I to CD8 T cells and class II to CD4 T cells, respectively (4). Importantly, CD8^+^ cDCs carry out the unique function of cross-presentation of exogenous antigens on class I molecules (2, 3). More recently, mouse cDC lineage has been further refined using expression history of DNGR-1 gene (9). Transfer of precursor DCs expressing DNGR-1 in mice depleted of myeloid cells leads to the development of cDCs but not to pDCs, as observed in the transfer of unfractionated precursor DCs.

In humans, myeloid cDCs can be categorized as CD1c^+^ (BDCA1^+^) and CD11a^+^ CD141^+^ (BDCA3^+^) DCs. The latter cells have been considered equivalent to mouse CD8^+^ DCs, particularly because they express the C-type lectin receptor CLEC9A, which mediates the uptake of necrotic or dead cells and the cross-presentation of the related antigens (10). However, recent evidence from a systematic study of DC populations showed that the functional specialization of human DCs is completely different from that of murine DCs (11). In contrast to the murine models, all human DC populations tested (BDCA1^+^ or BDCA3^+^ cDCs, and even BDCA2^+^ pDCs) express similar functions including cross-presentation and capacity of antigen transfer from phagoendosomes into the cytosol. In addition, Toll-like receptors (TLRs) are expressed differently by human and mouse cDC populations: both human and mouse cDCs express TLR1, 2, 3, 4, 5, 6, and 8, whereas TLR11, TLR12, and TLR13 are expressed only by mouse cDCs, and TLR 10 is unique to humans (12).

Plasmacytoid DCs represent a small fraction of DCs and have a round shape that is similar to antibody-secreting plasma cells. Regarding surface markers, pDCs are distinguished from cDCs by the expression of B220, Siglec-H, and Bst2 in mice and of BDCA2 (CD303) in humans (4). In both humans and mice, pDCs express TLR7 and 9 (13, 14). TLR7, 8, and 9 belong to a functional subfamily and detect PAMPs in endosomal/lysosomal compartments following acidification [reviewed in Ref. (15)]. After exposure to synthetic TLR7 or TLR9 agonists [e.g., imidazoquinoline compounds or guanosine analogs for TLR7/8, cytosine-phosphorothioate-guanine-oligodeoxynucleotides (CpG-ODNs) for TLR9], pDCs secrete interferon alpha and proinflammatory cytokines (IL-8 and tumor necrosis factor alpha) and undergo maturation, a differentiation program characterized by upregulation of the costimulatory molecules CD80, CD86, and CD40; expression of functional CC-chemokine receptor 7 (CCR7) and the maturation marker CD83; and heightened T-cell stimulatory capacity [reviewed in Ref. (15)]. The transcription factor E2-2 is essential for pDC development in both mice and humans (16). It controls the expression of pDC markers directly (e.g., TLR7, TLR9, BDCA2) and its deletion in mature pDCs, redirecting them toward the cDC phenotype. In contrast to cDCs, mouse pDCs are not phagocytic, and they maintain a high turnover of MHC class II, thus limiting their capacity as professional antigen-presenting cells (15).

More recently, a new DC subset defined as inflammatory DCs (infDCs) has been described in inflamed human tissues, including ascites of ovarian cancer (OC) and breast cancer (17). InfDCs (CD14^+^ CD16^−^ BDCA1^+^) in cancer ascites were separated from macrophages (CD14^+^ CD16c^+^ BDCA1^−^) and then further characterized for the expression of additional markers (CD11c^+^ CD11b^+^ HLA-DR^+^ BDCA1^+^ CD206^+^). InfDCs were identified in inflamed tissue but not in tumor-draining lymph nodes with the exception of gastric cancer, which is known to be associated with persistent chronic inflammation. Molecular profiling of the purified infDCs revealed a close similarity with monocyte-derived DCs. These cells induce a Th17 differentiation *in vitro* and express two lineage-specific transcription factors, ZBTB46 and CSFR1, which were previously identified in mouse infDCs. Functional assays showed that infDCs could stimulate memory CD4^+^ T cells from the same ascites to produce IL-17, likely by the secretion of IL-1β, IL-6, and IL-23, which are Th17 cell-polarizing cytokines (17).

Tumors can dramatically influence DC functions [reviewed in Ref. (6)]. It is well known that tumor-derived DCs are ineffective in stimulating an immune response and that this ineffectiveness may contribute to tumor evasion of immune recognition. Tumor-released factors can induce an altered myelopoiesis that leads to the release of immature myeloid cells, which, within the tumor bed, give rise to myeloid-derived suppressor cells (MDSCs). These findings, which have been confirmed in clinical studies, indicate a decreased presence and a defective functionality of mature DCs in patients with breast cancer (18), non-small cell lung cancer (19), pancreatic cancer (20), cervical cancer (21), hepatocellular carcinoma (22), and glioma (23). The fate of MDSCs has been investigated in various tumor types in relation to tumor drugs of different chemical nature including classical chemostatic agents, kinase inhibitors, and therapeutic antibodies. Pharmacological interventions, however, showed a marginal impact on DCs with respect to macrophages, which were skewed from an M2 (protumorigenic) toward an M1 (anti-tumor) phenotype. Differentiation toward proinflammatory DCs was induced by vascular endothelial growth factor inhibitors (24) or blockers of chemokines (25).

How the immune system senses tumors is not as well defined as for non-self-antigen recognition. The danger theory proposes that detection of stressed or damaged cells by DCs is a driving force of adaptive immune responses, irrespective of the level of mutation frequency of a given tumor (26).

## Cancer Therapy, DC Activation, Immune Responses, and Discovery of Tumor Antigens

**Figure 1:**
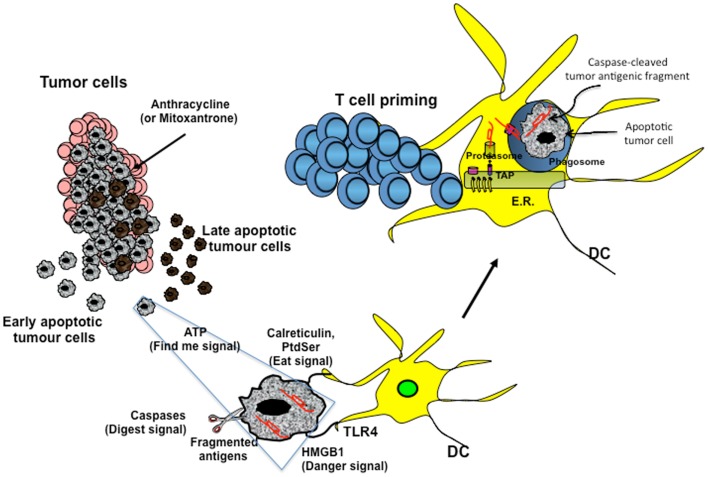
**Cross-presentation of chemotherapy-associated antigens derived from apoptotic tumor cells leading to anti-tumor immunity**. Tumor cells, upon chemotherapy treatment (i.e., anthracycline), undergo immunogenic cell death. According to the Kroemer–Zitvogel model, the immunogenic death (apoptosis, necrosis, autophagy, etc.) of cancer cells involves a multistep process, including the release of “find-me” signals (such as fractalkine, nucleotides, and ATP) that attract phagocytes or dendritic cells (DCs), the expression of “eat-me” signals [such as phosphatidylserine (PtdSer) and calreticulin] that facilitate recognition by phagocytes or DCs, and, finally, the release of danger-associated molecular patterns [such as high mobility group box 1 protein (HMGB1) and other signals described in the text] that enable dying tumor cells to lose the propensity to induce tolerance and to stimulate powerful anticancer immune responses. An additional factor that is involved in the success of the immunogenic chemotherapy may emerge from the capacity of caspases to cut and release apoptotic cell-associated antigenic fragments (in red in the figure), thus facilitating their transport from phagosomes into the cytosol and the processing by DCs (“digest-me” signals) via the class I-processing pathways [the figure emphasizes the model suggesting that caspase-cleaved apoptotic fragments are trimmed by cytosolic proteasomes in the form of peptides and that TAPs transport the resulting apoptotic self epitopes into the lumen of the endoplasmic reticulum (ER), where they can bind the appropiate class I molecule]. The final goal of these multiple checkpoints is to cross-present tumor epitopes and to elicit a wide repertoire of memory tumor-specific CD8^+^ T cells in patients undergoing tumor regression in response to appropriate chemotherapeutic regimes. These tumor-specific T cells represent a principal tool for discovering immunogenic tumor antigens by interrogating those responding to highly purified tumor proteins.

Cancer is inevitably treated with different drugs that vary either in the mechanism of action (ranging from the original chemostatic alkylating agents to pathway-specific inhibitors) or in their chemical nature (small-molecule drugs, neutralizing antibodies, cancer vaccines, etc.). The ever-growing arsenal of context-specific anti-tumor drugs is likely to be applied in unpredicted tumor cases thanks to technical progress in global genome sequencing, for which low prices have made it almost an affordable diagnostic approach. The most utilized therapeutic approaches, however, remain those based on cytotoxic chemotherapy. Although it is well known that these drugs induce lymphopenia, it is becoming more and more appreciated that a subset of them also induces a series of DAMPs, which are recognized by PRRs on innate immune cells (Figure 1).

Damage-associated molecular patterns such as ATP and high mobility group box 1 protein (HMGB1) are secreted or released, whereas others such as calreticulin (CRT) and heat shock protein 90 are exposed *de novo* or become enriched on the outer leaflet of the plasma membrane (27) (Figure 1). In addition, DAMPs are produced as end-stage degradation products such as uric acid during the course of cell death. Most of these molecules have predominantly non-immunological functions inside the cell before their exposure on the cell surface or their secretion (28).

The group of Kroemer and Zitvogel (29) found that treatment with anthracycline in mice induces immunogenic cell death (ICD), which is mediated by CRT exposure on apoptotic cells (Figure 1). Researchers have also reported that timing of CRT exposure with respect to apoptotic markers and morphological changes is critical during ICD and that it usually anticipates apoptotic signs (29, 30). This is a structured process that occurs through different pathways including RNA-dependent protein kinase-like endoplasmic reticulum kinase (PERK)-mediated eIF2α phosphorylation, the secretory pathway, and caspase 8-mediated B-cell receptor-associated protein 31 (BCAP31)-dependent activation of BAX and BAK proteins (31).

An additional DAMP signal released during ICD is the HMGB1 (Figure 1). Preclinical studies have highlighted the importance of TLR4 activation mediated by HMGB1 binding. Research has shown that depletion of HMGB1 in mouse xenograft tumors prevents anthracycline-induced anti-tumor activity, which is restored by exogenous recombinant HMGB1 protein. Clinical studies in breast cancer have showed that a correlation exists between the presence of a single nucleotide polymorphism in the TLR4 gene, which prevents the binding of HMGB1 to TLR4, and early relapse after anthracycline treatment (32, 33). However, the role of HMGB1 can be context-specific, depending on the oxidation state: reduced HMGB1 performs as a chemoattractant DAMP, whereas the fully oxidized form is inactive (34, 35).

A recent paper by Ma and colleagues (36) has identified the cellular mediator of ICD to be specific inflammatory DC-like cells in mice. In particular, monocytes recruited into the tumor bed skewed toward a DC phenotype, which includes expression of inflammatory DC markers (CD11c^+^CD11b^+^Ly6C^hi^). Tumors treated with mitoxantrone are infiltrated by CD11c^+^CD11b^+^ Ly6C^hi^ cells within 12 h of treatment and later by macrophages. This early infiltrate is responsible for the tumor-specific CD8^+^ T-cell response and anti-tumor activity, as these effects are abrogated by local expression of ATP-degrading enzyme CD39, pharmacological blockage of purinergic receptors, and neutralizing antibody against CD11. The extracellular release of ATP is used not only in different pathways, such as survival, death, adhesion, proliferation, differentiation, and mobility, but also as a “find-me signal” from apoptotic cells, which attract monocytes expressing purinergic receptors (Figure 1). Release of ATP during the apoptotic process is mediated by autophagy, which is induced by some chemotherapy treatments. Research has shown that knockdown of essential autophagy genes (ATG5, ATG7, and BECN1) reduces ATP secretion in apoptotic cells treated with anthracycline and results in reduced anti-tumor activity *in vivo* (37, 38). Apparently, ATP affects DCs by acting on two pathways. ATP, at a concentration of about 1 μM binds and activates P_2_Y_2_ receptor that induces monocyte attraction. At concentrations higher than 30 μM, ATP binds to P_2×7_ receptor and activates NALP3-ASC-inflammasome, inducing secretion of IL-1β, which skews antigen presentation to CD8^+^ T cells toward a Th-1 phenotype. The different activation threshold of P_2×7_ and P_2_Y_2_ receptors fits with a migratory/activation model, where low ATP concentrations in the periphery stimulate monocyte migration and higher ATP concentrations in the tumor bed induce DC differentiation.

The ICD concept presents new questions and challenges. Most of the mouse tumor models used to investigate ICD *in vivo* were based on tumor cell lines that did not evolve under an immunological pressure but rather were expanded *in vitro*. By contrast, the efficacy of immunogenic chemotherapy, such as the combination of oxaliplatin and doxorubicin, in spontaneous mouse tumor models has been shown to be independent of immune responses (39). Thus, it is important to determine the immunogenicity of anticancer therapies in humans and to identify which myeloid cells are recruited by chemotherapy.

Some evidence suggests that chemotherapy in humans is associated with antigen-specific immune responses (40, 41). Research on apoptotic antigens conducted previously in our laboratory has shown that caspase cleavage of self antigens derived from apoptotic cells facilitates their cross-presentation by DCs (42). Upon phagocytosis of apoptotic cells, the caspase-fragmented antigens can be efficiently exported by phagosomes into the cytosol, where they are processed through the class I-processing pathway and cross-presented in the form of peptides on class I molecules. In particular, self antigens, such as lamin B1, actin cytoplasmic 1, and vimentin, are normally sequestered in cell scaffolds; thus, they are unavailable for cross-presentation unless they are cleaved by caspases (43). The CD8^+^ T-cell responses to these epitopes are present in chronic viral infections including those caused by human immunodeficiency virus I and hepatitis C virus (42, 44), as well as in multiple sclerosis patients (45), and correlate with the disease progression.

To verify whether chemotherapy-induced apoptosis is immunogenic in humans, we analyzed OC patients who were treated with chemotherapy in the adjuvant setting (46) (Figure 1). To identify the immunogenic chemotherapy-associated antigens (CAAs), memory T cells from OC patients were interrogated with proteins isolated from primary OC cells by evaluating their response to two-dimensional electrophoresis gel-eluted OC proteins. Immunogenic CAAs were then molecularly characterized by mass spectrometry (MS)-based analysis. Memory T-cell responses against CAAs derived from apoptotic (but not live) OC cells correlated with prolonged survival in response to chemotherapy, thereby supporting the model of chemotherapy-induced apoptosis as an adjuvant of anti-tumor immunity (46). In addition, memory CD8^+^ T cells specific for individual OC proteins were elicited upon cross-presentation of CAAs or whole apoptotic (but not live) OC cells, suggesting that cross-presentation of tumor antigens and T-cell responses could contribute to the efficacy of anticancer chemotherapy. The antigen-specific CD4^+^ and CD8^+^ T-cell responses that were originally observed in the screening with proteins extracted from primary cancer cells were further confirmed using corresponding recombinant proteins. It is interesting to note that antigen-specific CD4^+^ and CD8^+^ T cells produced either IFN-γ or IL-17, which is in line with the recently described Th17 cell-polarizing infDCs described in human OC cases (17). MS-based analysis of CAAs showed enrichment for proteins of stress pathways such as Ras-related protein, heat shock protein β1, and heat shock protein α-B-crystallin. Taken together, these data suggest that CAAs correspond not necessarily to tumor cell-specific antigens but rather to ubiquitous proteins, which, under normal conditions, are sequestered in cell structures that limit their processing and presentation to T cells. However, as a result of the chemotherapy effects, apoptosis of tumor cells can induce upregulation of a wide range of ubiquitous proteins sufficient for subsequent processing and presentation by DCs, which in turn could prime the corresponding specific T cells (Figure 1).

There are many open questions surrounding how DCs can drive immune responses during chemotherapy in humans and whether the memory immune response against CAAs plays any role in preventing tumor relapses. We believe that the identification of new immune correlates can help in refining a more targeted and effective anticancer therapy.

## Author Contributions

Fabio Palombo conducted the literature review and cowrote the manuscript, Chiara Focaccetti helped in finalizing the manuscript, and Vincenzo Barnaba provided overall supervision and cowrote the manuscript.

## Conflict of Interest Statement

The authors declare that the research was conducted in the absence of any commercial or financial relationships that could be construed as a potential conflict of interest.
